# Maternal Age at Holocaust Exposure and Maternal PTSD Independently Influence Urinary Cortisol Levels in Adult Offspring

**DOI:** 10.3389/fendo.2014.00103

**Published:** 2014-07-04

**Authors:** Heather N. Bader, Linda M. Bierer, Amy Lehrner, Iouri Makotkine, Nikolaos P. Daskalakis, Rachel Yehuda

**Affiliations:** ^1^James J. Peters VA Medical Center, Bronx, NY, USA; ^2^Department of Psychiatry, Icahn School of Medicine at Mount Sinai, New York, NY, USA

**Keywords:** maternal, PTSD, risk, cortisol, intergenerational, Holocaust, offspring, trauma

## Abstract

**Background:** Parental traumatization has been associated with increased risk for the expression of psychopathology in offspring, and maternal posttraumatic stress disorder (PTSD) appears to increase the risk for the development of offspring PTSD. In this study, Holocaust-related maternal age of exposure and PTSD were evaluated for their association with offspring ambient cortisol and PTSD-associated symptom expression.

**Method:** Ninety-five Holocaust offspring and Jewish comparison subjects received diagnostic and psychological evaluations, and 24 h urinary cortisol was assayed by RIA. Offspring completed the parental PTSD questionnaire to assess maternal PTSD status. Maternal Holocaust exposure was identified as having occurred in childhood, adolescence, or adulthood and examined in relation to offspring psychobiology.

**Results:** Urinary cortisol levels did not differ for Holocaust offspring and comparison subjects but differed significantly in offspring based on maternal age of exposure and maternal PTSD status. Increased maternal age of exposure and maternal PTSD were each associated with lower urinary cortisol in offspring, but did not exhibit a significant interaction. In addition, offspring PTSD-associated symptom severity increased with maternal age at exposure and PTSD diagnosis. A regression analysis of correlates of offspring cortisol indicated that both maternal age of exposure and maternal PTSD were significant predictors of lower offspring urinary cortisol, whereas childhood adversity and offspring PTSD symptoms were not.

**Conclusion:** Offspring low cortisol and PTSD-associated symptom expression are related to maternal age of exposure, with the greatest effects associated with increased age at exposure. These effects are relatively independent of the negative consequences of being raised by a trauma survivor. These observations highlight the importance of maternal age of exposure in determining a psychobiology in offspring that is consistent with increased risk for stress-related pathology.

## Introduction

Parental posttraumatic stress disorder (PTSD) has been identified as a salient contributor to the transgenerational transmission of risk for low cortisol trait and the development of PTSD in offspring (1–4). Furthermore, parental traumatization has been associated with increased risk for offspring depression and anxiety disorders (5, 6). Recent studies have noted the specific association of maternal, rather than paternal PTSD with lifetime expression of offspring PTSD has been previously noted (7) and linked maternal PTSD and even depression following exposure to the World trade center attacks to increased emotional reactivity in child offspring (8). The majority, but not all, of the studies have been done in Holocaust offspring (8). The Holocaust offspring population offers numerous advantages for the study of intergenerational effects including the opportunity to evaluate consequences of age of exposure, which has not previously been the focus of study. Because the Holocaust was a discrete historical event, persons of different ages were exposed simultaneously, allowing an investigation of the effect of age of trauma exposure on offspring symptoms and biology. Thus, although maternal PTSD has been identified as a strong contributor to hypothalamic–pituitary–adrenal (HPA) axis sensitivity and offspring psychopathology (7–10), the impact of maternal age at Holocaust exposure on offspring biology has not been sufficiently investigated as a potential contributor. In this report, we examine urinary cortisol levels in Holocaust survivor offspring and comparison subjects in relation to maternal age of Holocaust exposure and PTSD by presenting a reanalysis of previously reported data (1), with the addition of new subjects. The Holocaust offspring in this study was conceived after, and in some cases well after, direct parental exposure to the Holocaust. Thus, this is an optimal sample in which to evaluate effects in offspring related to age of maternal exposure, as well as to maternal PTSD.

We hypothesized lower cortisol and greater PTSD symptomatology in offspring of mothers who were adults during their Holocaust exposure based on previous observations that older survivors had greater distressing thoughts and nightmares, contributing to greater PTSD symptoms than younger survivors (11). This would cause greater glucocorticoid disturbances in the survivor and potentially also a more adverse post-natal environmental effect on the offspring.

## Materials and Methods

### Subjects

Ninety-five participants, containing Holocaust survivor offspring (*n* = 69) and Jewish comparison subjects (*n* = 26), were included in the current study. Data from 31 of the Holocaust survivor offspring and 13 controls was previously published (1). Participants were recruited primarily through advertisements seeking Jewish volunteers and through Holocaust survivors who had previously participated in related research and were made aware of the current study as previously described (1, 2). Participant written, informed consent, and Icahn School of Medicine at Mount Sinai Institutional Review Board approval were received for all study procedures.

Holocaust survivor offspring was defined as having been born after World War II (1944 or later) and raised through adolescence by birth parents that were both exposed to the Nazi Holocaust. Comparison subjects were demographically comparable Jewish persons born to parents unexposed to the Holocaust, generally from the United States or Canada. Participants with psychotic illness, bipolar disorder, obsessive compulsive disorder, or a medical illness or medication that might interfere with HPA axis function were not studied.

Psychological evaluations were administered to assess the presence of any current or lifetime psychiatric disorders, including PTSD, according to DSM-IV criteria using the Structured Clinical Interview for the DSM-IV (12) and Clinician-Administered PTSD Scale [CAPS; (13)], respectively. Other self-rating instruments assessed depression and anxiety severity [Beck Depression Inventory (BDI); (14)] and Spielberger State-Trait Anxiety Inventory (STAI); (15), and severity of childhood adversity [Childhood Trauma Questionnaire (CTQ); (16)]. Additionally, Holocaust offspring was asked to rate parental symptoms using the Parental PTSD Questionnaire (PPQ), an instrument that has been previously developed in our laboratory and has demonstrated high concordance between offspring and clinician-rated evaluations of Holocaust survivors (17). The scale further inquires about potential consequences of being raised by a Holocaust survivor; for instance, becoming more or less harm avoidant, experiencing “psychological scars,” or developing increased “stress sensitivity” (17). Other information about parents such as year of birth, age at Holocaust exposure, type of Holocaust exposure (e.g., concentration camp, hiding), and age at offspring birth, was also determined. The Holocaust offspring group was then further subcategorized based on maternal age of exposure and the presence or absence of maternal PTSD, as recently described (18).

### Urine collection and cortisol assay

Participants were asked to collect 24-h urinary samples at home on a day anticipated to be relatively unstressful and in which vigorous exercise was avoided. Completeness of collection was monitored by asking participants about missed collections as well as by assessing urinary creatinine concentrations. The laboratory personnel who performed the assays were blind to whether the sample came from a Holocaust survivor offspring or comparison subject as well as any demographical or clinical information about the subjects who provided the samples. More detailed procedures for 24-h urine collections, storage, and cortisol determination have been described previously (2).

### Statistical methods

The purpose of the analyses was to compare Holocaust offspring to controls, or to examine differences between Holocaust offspring subgroups based maternal age of exposure (11 years or younger, 12–18 years of age, 18 years or older) and/or presence or absence of maternal PTSD. Other analyses examined associations between clinical and descriptive measures. Chi-square analyses were used to compare categorical variables, and analyses of variance and covariance (ANOVA, ANCOVA) were used to compare descriptive data across groups. Covariates were detected by performing correlations between urinary cortisol and other outcome measures with body mass index (BMI), presence/absence of current depressive disorder diagnosis, medication usage, and other variables known to influence the HPA axis; variables were included as covariates in analyses involving outcome measures with which they were significantly correlated. Age and gender are used as covariates in analyses including all subjects as there is an established biological basis for their influence on urinary cortisol (19, 20). Significance was set at *p* < 0.05 and trend level significance was set at *p* < 0.10. Bonferroni *post hoc* testing was performed when applicable.

## Results

### Comparison of Holocaust offspring and controls

Table 1 compares demographical and clinical characteristics, as well as urinary cortisol levels, for Holocaust offspring and Jewish comparison subjects. The groups differed significantly on age, presence of current and lifetime anxiety disorder as well as self-ratings of anxiety. There were trends for differences in self-reported and diagnosis of current depression and lifetime PTSD diagnosis, as well as childhood trauma. Furthermore, there was no significant difference between comparison subjects and Holocaust offspring urinary cortisol levels (*F*_1,91_ = 0.72, ns; covaried for age and gender).

**Table 1:** **Comparison of Holocaust offspring and comparison subjects**.

	Offspring (*n* = 69)	Control (*n* = 26)	*F*_df_, *p*, or $\chi_{\text{df}}^{2}$, *p*
Age	47.9 ± 7.4	42.5 ± 10.7	*F*_1,93_ = 7.70, *p* = 0.007
Gender (% males)	23 (33.3%)	12 (46.2%)	$\chi_{1}^{2}$ = 1.33, ns
Years of education	17.2 ± 3.1	16.9 ± 2.2	*F*_1,93_ = 0.17, ns
Body mass index (kg/m^2^)	24.6 ± 4.3	24.1 ± 4.0	*F*_1,93_ = 0.25, ns
Beck Depression Inventory	8.1 ± 6.9	4.4 ± 5.6	*F*_1,64_ = 3.96, (*p* = 0.051)
Spielberger Trait Anxiety^a^	21.4 ± 10.9	15.1 ± 11.5	*F*_1,60_ = 4.21, *p* = 0.044
Spielberger State Anxiety^a^	16.8 ± 12.6	9.4 ± 8.8	*F*_1,64_ = 5.09, *p* = 0.028
CTQ total score^b^	41.2 ± 13.6	35.9 ± 12.1	*F*_1,87_ = 2.88, (*p* = 0.094)
CAPS total score – current^c^	14.8 ± 20.8	8.7 ± 19.7	*F*_1,85_ = 1.46, ns
CAPS total score – lifetime^c^	24.5 ± 26.5	15.9 ± 25.7	*F*_1,86_ = 1.86, ns
Depressive disorder – current^d^	13 (18.8%)	1 (3.8%)	$\chi_{1}^{2}$ = 3.38, (*p* = 0.066)
Depressive disorder – lifetime^d^	32 (46.4%)	8 (30.8%)	$\chi_{1}^{2}$ = 1.89, ns
Anxiety disorder – current^d^	22 (31.9%)	3 (11.5%)	$\chi_{1}^{2}$ = 4.03, *p* = 0.045
Anxiety disorder – lifetime^d^	32 (46.4%)	5 (19.2%)	$\chi_{1}^{2}$ = 5.85, *p* = 0.016
PTSD – current^e^	4 (5.8%)	1 (4.0%)	$\chi_{1}^{2}$ = 0.12, ns
PTSD – lifetime^e^	13 (18.8%)	1 (4.0%)	$\chi_{1}^{2}$ = 3.19, (*p* = 0.074)
Urinary cortisol (RIA; μg/day)	51.3 ± 27.5	57.6 ± 31.4	*F*_1,93_ = 0.90, ns

*^a^Spielberger State-Trait Anxiety Inventory (STAI); ^b^Childhood Trauma Questionnaire (CTQ); ^c^Clinician-Administered PTSD Scale (CAPS) ratings for PTSD-related symptom severity; ^d^Diagnoses according to DSM-IV criteria based on clinical interview; ^e^Diagnoses based on CAPS criteria for current and lifetime PTSD*.

### Holocaust offspring according to maternal age at exposure

Table 2 compares demographical and clinical characteristics for Holocaust offspring according to maternal age at exposure. As expected because the Holocaust occurred during a circumscribed period in history, the groups differed significantly on offspring age and maternal age at offspring birth. There were also significant differences between offspring groups on self-reported psychological scars and stress sensitivity, and PTSD severity, which were confirmed by *post hoc* testing to reflect significant differences between offspring with maternal exposures as children versus adults.

**Table 2:** **Holocaust offspring variables according to maternal age at exposure**.

	Maternal age at Holocaust exposure			F_df_, *p*, or $\chi_{\text{df}}^{2}$, *p*
	Child (*n* = 22)	Adolescent (*n* = 30)	Adult (*n* = 17)	
Offspring age	43.0 ± 8.0	48.6 ± 6.1	52.8 ± 4.4	*F*_2,66_ = 11.47, *p* < 0.0005,b^a,b^
Maternal age at offspring birth	26.6 ± 5.5	29.4 ± 4.6	35.0 ± 4.7	*F*_2,61_ = 12.50, *p* < 0.0005,c^b,c^
Gender (% males)	6 (27.3%)	10 (33.3%)	7 (41.2%)	$\chi_{2}^{2}$ = 0.83, ns
Years of education	17.5 ± 3.4	16.9 ± 3.2	17.3 ± 2.5	*F*_2,66_ = 0.20, ns
Body mass index (kg/m^2^)	23.9 ± 3.3	25.0 ± 4.5	24.7 ± 5.4	*F*_2,66_ = 0.35, ns
Psychological scars and stress sensitivity^d^	4.9 ± 2.9	5.9 ± 2.5	7.4 ± 2.6	*F*_2,61_ = 3.63, *p* = 0.032^b^
Beck Depression Inventory	8.7 ± 6.7	7.0 ± 5.8	9.3 ± 9.1	*F*_2,45_ = 0.52, ns
Spielberger STAI-T^e^	20.4 ± 9.7	21.6 ± 10.4	22.5 ± 13.9	*F*_2,41_ = 0.11, ns
Spielberger STAI-S^e^	16.5 ± 11.4	15.5 ± 13.7	19.2 ± 12.8	*F*_2,45_ = 0.31, ns
CTQ total score^f^	41.1 ± 15.0	41.2 ± 12.7	41.4 ± 14.5	*F*_2,61_ = 0.00, ns
CAPS total score – current^g^	8.0 ± 15.6	14.0 ± 20.7	25.3 ± 24.1	*F*_2,61_ = 3.23, *p* = 0.046^b^
CAPS total score – lifetime^g^	14.5 ± 21.2	23.8 ± 25.3	39.2 ± 29.9	*F*_2,61_ = 4.11, *p* = 0.021^b^
Depressive disorder – current^h^	5 (22.7%)	4 (13.3%)	4 (23.5%)	$\chi_{2}^{2}$ = 1.06, ns
Depressive disorder – lifetime^h^	8 (36.4%)	15 (50.0%)	9 (52.9%)	$\chi_{2}^{2}$ = 1.34, ns
Anxiety disorder – current^h^	6 (27.3%)	11 (36.7%)	5 (29.4%)	$\chi_{2}^{2}$ = 0.58, ns
Anxiety disorder – lifetime^h^	7 (31.8%)	16 (53.3%)	9 (52.9%)	$\chi_{2}^{2}$ = 2.75, ns
PTSD – current^i^	1 (4.5%)	1 (3.3%)	2 (11.8%)	$\chi_{2}^{2}$ = 1.51, ns
PTSD – lifetime^i^	3 (13.6%)	5 (16.7%)	5 (29.4%)	$\chi_{2}^{2}$ = 1.73, ns
Urinary cortisol (RIA; μg/day)	57.8 ± 25.3	52.7 ± 28.2	40.6 ± 27.5	*F*_2,66_ = 1.98, ns*^j^*

*^a^Significant *post hoc* difference between offspring of child and adolescent survivors; ^b^Significant *post hoc* difference between offspring of child and adult survivors; ^c^Significant *post hoc* difference between offspring of adolescent and adult survivors; ^d^Composite score of two self-report items on the Parental PTSD Questionnaire (PPQ); ^e^Spielberger State-Trait Anxiety Inventory (STAI); ^f^Childhood Trauma Questionnaire (CTQ); ^g^Clinician-Administered PTSD Scale (CAPS) ratings for PTSD-related symptom severity; ^h^Diagnoses according to DSM-IV criteria based on clinical interview; ^i^Diagnoses according to CAPS criteria for current and lifetime PTSD*.

*^j^Comparison without covariates as used in text*.

**Figure 1:**
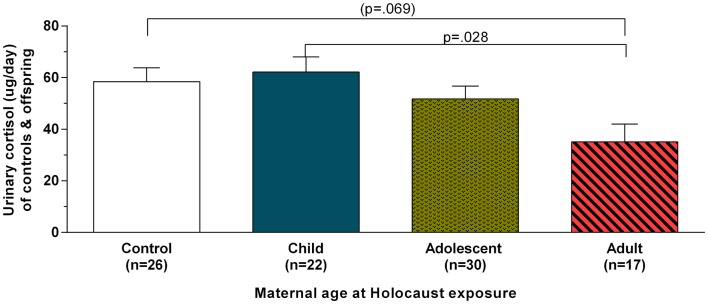
**Twenty-four-hour urinary cortisol excretion based on maternal age at Holocaust exposure in control subjects and Holocaust offspring**. Estimated marginal means ± SEM for urinary cortisol levels (microgram per day) are presented for comparison subjects (white bar) and Holocaust survivor offspring grouped according to whether offspring’s mother was a child (age 0–11; solid blue bar), adolescent (age 12–18; speckled gold bar), or adult (diagonally striped red bar) at Holocaust exposure (age 18 or older). Statistical significance was set at *p* < 0.05.

**Figure 2:**
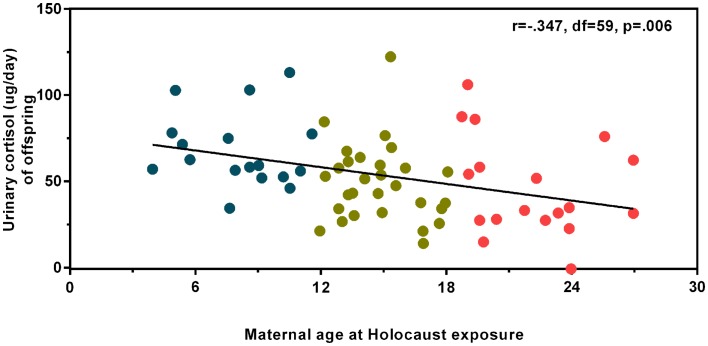
**Relationship of maternal age at Holocaust exposure with offspring 24-h urinary cortisol excretion**. A partial correlation between maternal age at Holocaust exposure and Holocaust offspring cortisol level controlling for offspring age, gender, and current depressive disorder diagnosis (*r* = −0.347, d*f* = 59, *p* = 0.006) is depicted using linear regression and unstandardized residuals that were added to the raw values. Data points are colored for ease of interpretation to associate with the child (blue), adolescent (gold), and adult (red) maternal age of exposure groups. The total number of subjects included in this analysis is 64. The correlation coefficients are denoted and the statistical significance was set at *p* < 0.05.

Figure 1 illustrates urinary cortisol levels for controls and offspring grouped by maternal age at Holocaust exposure. The main effect of group (*F*_3,89_ = 3.09, *p* = 0.031; covaried for age, and gender) resulted from a significant difference between offspring whose mothers were adults and those whose mothers were children at time of exposure (*p* = 0.028) and a trend level difference between offspring whose mothers were exposed during adulthood and controls (*p* = 0.069). There was no significant difference between offspring with mothers exposed during childhood compared to those with mothers exposed during adolescence or between controls, between either of the latter two groups, or between adult and adolescent maternally exposed offspring. Figure 2 illustrates that strength of the association between maternal age at exposure and urinary cortisol levels when age was expressed as a continuous variable for Holocaust offspring only, covaried for age, gender, and current depressive disorder (*r* = −0.35, d*f* = 59, *p* = 0.006). This relationship also remained significant when additionally accounting for offspring subjective assessment of childhood adversity (*r* = −0.29, d*f* = 59, *p* = 0.006).

The observed relationship of offspring urinary cortisol with maternal age of exposure was not driven by maternal age at offspring birth. The relationship between urinary cortisol and maternal age at offspring birth in Holocaust offspring was not significant (*r* = −0.206, d*f* = 59, *p* = 0.112, controlling for offspring age, gender, and current depressive disorder) and this relationship was unchanged when maternal age at Holocaust was added as a covariate (*r* = 0.162, d*f* = 58, *p* = 0.217). Maternal age at offspring birth was also not significantly associated with urinary cortisol levels in the control group (*r* = 0.274, df = 15, *p* = 0.287).

### Holocaust offspring according to maternal PTSD

Table 3 shows demographic and clinical characteristics of Holocaust offspring only by maternal PTSD status. As previously reported for a subset of this group, in this larger sample, offspring with maternal PTSD reported significantly greater psychological scars and stress sensitivity, were diagnosed more frequently with lifetime PTSD, and showed higher levels of anxiety and PTSD-associated symptoms. Offspring with maternal PTSD also demonstrated trends for increased self-ratings of depression, state anxiety, and childhood trauma exposure. There was a significant main effect of maternal PTSD on Holocaust offspring urinary cortisol (*F*_1,63_ = 6.35, *p* = 0.014; covaried for age, gender, and current depressive disorder).

**Table 3:** **Holocaust offspring variables according to maternal PTSD status**.

	Maternal PTSD status		*F*_df_, *p* or $\chi_{\text{df}}^{2}$, *p*
	PTSD+ (*n* = 42)	PTSD− (*n* = 26)	
Offspring age	47.6 ± 7.5	48.7 ± 7.1	*F*_1,66_ = 0.41, ns
Maternal age at offspring birth	29.6 ± 5.2	29.7 ± 6.5	*F*_1,61_ = 0.01, ns
Gender (males)	16 (38.1%)	7 (26.9%)	$\chi_{1}^{2}$ = 0.90, ns
Number of years of education	17.2 ± 2.7	17.1 ± 3.7	*F*_1,66_ = 0.04, ns
Body mass index (kg/m^2^)	24.7 ± 4.1	24.4 ± 4.9	*F*_1,66_ = 0.07, ns
Psychological scars and stress sensitivity^a^	6.8 ± 2.7	4.6 ± 2.4	*F*_1,62_ = 11.70, *p* = 0.001
Beck Depression Inventory	9.6 ± 7.9	6.3 ± 5.2	*F*_1,46_ = 2.88, *p* = 0.097
Spielberger STAI-T^b^	25.5 ± 10.2	16.1 ± 9.7	*F*_1,42_ = 9.77, *p* = 0.003
Spielberger STAI-S^b^	19.6 ± 12.8	13.1 ± 11.7	*F*_2,46_ = 3.20, *p* = 0.080
CTQ total score^c^	46.8 ± 13.9	32.2 ± 7.1	*F*_1,61_ = 22.64, *p* < 0.0005
CAPS total score – current^d^	19.6 ± 24.5	6.7 ± 9.7	*F*_1,61_ = 6.04, *p* = 0.017
CAPS total score – lifetime^d^	31.7 ± 29.5	11.9 ± 14.6	*F*_1,61_ = 9.42, *p* = 0.003
Depressive disorder – current^e^	10 (23.8%)	3 (11.5%)	$\chi_{1}^{2}$ = 1.56, ns
Depressive disorder – lifetime^e^	23 (54.8%)	9 (34.6%)	$\chi_{1}^{2}$ = 2.62, ns
Anxiety disorder – current^e^	10 (23.8%)	11 (42.3%)	$\chi_{1}^{2}$ = 2.67, ns
Anxiety disorder – lifetime^e^	17 (40.5%)	14 (53.8%)	$\chi_{1}^{2}$ = 1.16, ns
PTSD – current^f^	4 (9.5%)	0 (0.0%)	$\chi_{1}^{2}$ = 2.63, ns
PTSD – lifetime^f^	12 (28.6%)	1 (3.8%)	$\chi_{1}^{2}$ = 6.35, *p* = 0.012
Urinary cortisol (RIA; μg/day)	46.7 ± 23.7	59.3 ± 32.1	*F*_1,66_ = 3.42, (*p* = 0.069)^g^

*^a^Composite score of two self-report items on the Parental PTSD Questionnaire (PPQ); ^b^Spielberger State-Trait Anxiety Inventory (STAI); ^c^Childhood Trauma Questionnaire (CTQ); ^d^Clinician-Administered PTSD Scale (CAPS) ratings for PTSD-related symptom severity; ^e^Diagnoses according to DSM-IV criteria based on clinical interview; ^f^Diagnoses according to CAPS criteria for current and lifetime PTSD; ^g^Comparison made without covariates as in text*.

### Offspring cortisol in relation to maternal age at exposure and PTSD

**Figure 3:**
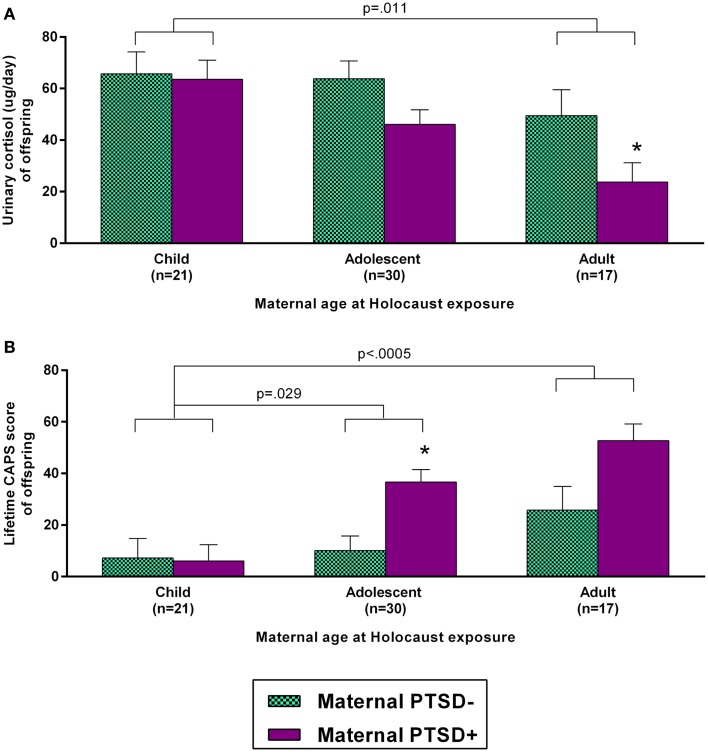
**Influence of maternal age at Holocaust exposure and maternal PTSD on offspring 24-h urinary cortisol excretion and lifetime PTSD-related symptom severity**. Offspring cortisol **(A)** and offspring lifetime clinician-administered PTSD scale (CAPS) total score **(B)** are depicted based on maternal age at Holocaust and maternal PTSD status. Maternal PTSD − offspring are depicted with light blue, checkered bars and maternal PTSD + offspring are represented with solid purple bars. Data were adjusted for age, gender, and diagnosis of current depressive disorder and are represented as estimated marginal means ± SEM. *, versus PTSD – was statistically significant. Statistical significance was set at *p* < 0.05.

As both maternal age at exposure and PTSD were associated with offspring urinary cortisol, these predictors were entered as main effects in a single ANCOVA including only Holocaust offspring, controlling for offspring age, gender, and current depressive disorder. Both main effects were significant (maternal age at exposure: *F*_2,59_ = 4.76, *p* = 0.012; maternal PTSD: *F*_1,59_ = 5.85, *p* = 0.019) but their interaction was not (*F*_2,59_ = 1.12, ns). All covariates were significant in this model (age, *p* = 0.015; gender, *p* = 0.014; current depressive disorder, *p* = 0.008). These data are depicted in Figure 3A. Adding childhood trauma as a covariate, reduced the main effect of maternal PTSD to a trend. Figure 3B demonstrates that a similar analysis examining lifetime offspring PTSD-associated symptom severity also showed a significant effect of maternal age at exposure (*F*_2,54_ = 8.46, *p* = 0.001) and maternal PTSD status (*F*_2,54_ = 9.87, *p* = 0.003); covaried for offspring age, gender, and current depressive disorder. Offspring whose mothers were children during their Holocaust exposure had significantly lower PTSD-associated symptom severity than those whose mothers were adults (*p* < 0.0005) or adolescents (*p* = 0.029). Adding childhood trauma severity as a covariate reduced the effect of maternal PTSD such that it was no longer significant. Similar results were found for current offspring PTSD-related symptom severity and PPQ assessment of perceived psychological scars and stress sensitivity.

### Impact of maternal and offspring characteristics on urinary cortisol excretion

A regression analysis was performed to examine the relative contributions of maternal and offspring characteristics on Holocaust offspring cortisol. As a first step, age, gender, and presence of current depressive disorder were entered. Maternal age at Holocaust exposure, presence or absence of maternal PTSD, childhood trauma severity, and offspring PTSD-associated symptom severity (lifetime CAPS scores) were added as a second step. After controlling for variables in the first step, and each of the four variables in the second step, only maternal age at Holocaust exposure (β = −0.562, *p* = 0.001) and maternal PTSD (β = −0.317, *p* = 0.020) remained significant (adjusted *R*^2^ = 0.361, adjusted Δ*R*^2^ = 0.218), but offspring PTSD and childhood trauma were not significant.

## Discussion

The results of the study replicate and extend previous findings by our group (1, 2, 9, 21) regarding cortisol levels in Holocaust offspring. It has been previously established that parental, and specifically maternal, PTSD associate with lower cortisol levels in offspring even after accounting for offspring’s own traumatization and PTSD (1, 2, 5, 7, 9). This study adds that the low cortisol levels in association with maternal PTSD are additionally associated with maternal age at Holocaust exposure. In fact, maternal age at Holocaust exposure appears to exert a parallel effect on cortisol in offspring that is not a result of maternal PTSD or an interaction with maternal PTSD. The lowest levels of urinary cortisol were present in offspring of mothers with PTSD who survived the Holocaust as adults.

In parallel, maternal age at Holocaust exposure and presence of maternal PTSD were found to be similarly associated with psychological characteristics, particularly increased PTSD-related symptoms reflected by the CAPS total score, which may also reflect perceived psychological scars and increased stress sensitivity as a result of being raised by Holocaust survivors. This conclusion is supported by literature that demonstrates that offspring of trauma survivors have increased vulnerability to distress (22). The influence of maternal PTSD was also associated with increased scores on both of these psychological measures. However, when all variables, including childhood adversity, which in offspring is in part a function of having Holocaust survivor parents (with or without PTSD), were included in a regression analysis, maternal age at exposure, and maternal PTSD appeared to be independent contributors to offspring cortisol and symptoms. These maternal variables were more strongly associated with offspring urinary cortisol excretion than were the offspring’s own PTSD-associated symptoms or the related feelings of being victimized by their parents’ exposures, interpreted as being psychologically scarred or having been sensitized to stress.

In that low cortisol has been associated with the PTSD diagnosis in Holocaust survivors (23), and indeed in PTSD related to other exposures (24–28), it is noteworthy that offspring’s own PTSD symptom severity was less associated with low cortisol levels than the parental risk factors described above. We have previously demonstrated lower cortisol levels in offspring samples with no PTSD and have suggested that low cortisol levels may be related to PTSD vulnerability, potentially due to the presence of parental PTSD acting as a risk factor (1, 2, 5, 7, 9). In this sample, only 12/69 offspring met diagnostic criteria for lifetime PTSD and only 4 met diagnostic criteria for current PTSD. Thus, the assessment of PTSD severity using the CAPS score reflects relatively low levels of symptom severity in this sample. Possibly, this accounts for the relative lack of association of low cortisol with PTSD once other risk factors are considered.

The association of low cortisol in offspring with maternal age of exposure in addition to maternal PTSD implies an intergenerational effect on offspring that does not result from maternal PTSD symptoms *per se*. Studies of other populations have provided information that the “intergenerational effects” on cortisol are manifest in offspring at a very young age, including during infancy, as was demonstrated in infants of mothers with PTSD exposed to the 9/11 World Trade Center attacks while pregnant (29). The significant trimester effect observed in that study suggest some role for the intrauterine environment in transmission (30). In the current study, all offspring were born after the Holocaust had ended, and any potential intrauterine effects were not a result of direct *in utero* exposure.

The question raised by the data concern potential differences in intergenerational transmission depending on maternal developmental or age-related factors during trauma exposure. A previous study demonstrated that Holocaust survivors tended to show slightly different symptom profiles depending on whether they were children or adults during the war. Holocaust survivors who were younger at the age of Holocaust exposure had a higher prevalence of hypervigilance, psychogenic amnesia, and emotional detachment, while survivors who were older at time of exposure experience more distressing intrusive thoughts and nightmares (11). It stands to reason that if symptom profiles associate with age of exposure, biological characteristics might also associate with age of exposure.

In fact, the activity of kidney 11β-hydroxysteroid dehydrogenase type 2 (11β-HSD-2) enzyme, which converts active cortisol to inert cortisone, was found to be higher in older Holocaust survivors (31), who displayed lower cortisol reactivity (32). Interestingly, in the current Holocaust offspring sample, we observed that 11β-HSD-2 activity was higher in offspring whose mothers were younger at the time of exposure (18). Thus, in that the intergenerational effect on 11β-HSD-2 was in the opposite direction in offspring compared with survivors, the effect in offspring may have in some measure obviated the consequences associated with PTSD risk in the form of low cortisol and symptoms. Thus, the current finding of lower cortisol and greater symptoms in association with older maternal exposure may reflect the absence of a protective epigenetic accommodation associated with the experience of extreme trauma in childhood, a particularly vulnerable period for developmental programing (30, 33, 34), in the Holocaust survivor parent.

The results of this study have a broad and enduring relevance, as they demonstrate not only potential psychological effects, but also a biological effect of maternal trauma exposure on unexposed offspring. Thus, distinct phenotypes may be observed in the offspring based not only on maternal experiences and the development of PTSD as a consequence, but also on the time of life during which a mother endured traumatic experiences. The study underscores prior observations that distinct phenotypes may result from trauma exposure at different ages (11), and further notes the potential for differential consequences for transmission in the next generation. Additional investigation is required to explore the possibility that these effects may span generations beyond the direct offspring of trauma survivors and explore potential mechanisms of this transmission.

## Author Contributions

Rachel Yehuda designed the study. Rachel Yehuda and Linda M. Bierer supervised the project and data collection. Linda M. Bierer supervised the clinical assessments, and Rachel Yehuda supervised the biological collection and protocol. Iouri Makotkine performed the biological assays and quality control. Heather N. Bader did primary analyses and drafted the manuscript. Nikolaos P. Daskalakis and Amy Lehrner assisted with literature review, manuscript preparation and editing. Rachel Yehuda and Linda M. Bierer edited the final manuscript. All the authors discussed the results and commented on the final version of the manuscript.

## Conflict of Interest Statement

The authors declare that the research was conducted in the absence of any commercial or financial relationships that could be construed as a potential conflict of interest.
